# Distinct Dasatinib-Induced Mechanisms of Apoptotic Response and Exosome Release in Imatinib-Resistant Human Chronic Myeloid Leukemia Cells

**DOI:** 10.3390/ijms17040531

**Published:** 2016-04-08

**Authors:** Juan Liu, Yujing Zhang, Aichun Liu, Jinghua Wang, Lianqiao Li, Xi Chen, Xinyu Gao, Yanming Xue, Xiaomin Zhang, Yao Liu

**Affiliations:** 1 Department of Hematology, Harbin Medical University Second Hospital, Harbin 150086, China; liujuancheng79@163.com (J.L.); jinghua_wang57@163.com (J.W.); chenxihan78@163.com (X.C.); gaoxinyujiang83@163.com (X.G.); xueyanming82@163.com (Y.X.); zhangxiaominli81@163.com (X.Z.); liuyaoliu87@163.com (Y.L.); 2 Department of Pediatrics, Harbin Medical University Second Hospital, Harbin 150086, China; zhangyujing73@gmail.com; 3 Department of Hematology and Lymphology, Harbin Medical University Cancer Hospital, Harbin 150086, China; lilianqiao85@126.com

**Keywords:** Imatinib-resistant K562, mTOR, autophagy, apoptosis, exosome, dasatinib

## Abstract

Although dasatinib is effective in most imatinib mesylate (IMT)-resistant chronic myeloid leukemia (CML) patients, the underlying mechanism of its effectiveness in eliminating imatinib-resistant cells is only partially understood. This study investigated the effects of dasatinib on signaling mechanisms driving-resistance in imatinib-resistant CML cell line K562 (K562R^IMT^). Compared with K562 control cells, exsomal release, the phosphoinositide 3-kinase (PI3K)/protein kinase B (Akt)/ mammalian target of rapamycin (mTOR) signaling and autophagic activity were increased significantly in K562R^IMT^ cells and mTOR-independent beclin-1/Vps34 signaling was shown to be involved in exosomal release in these cells. We found that Notch1 activation-mediated reduction of phosphatase and tensin homolog (PTEN) was responsible for the increased Akt/mTOR activities in K562R^IMT^ cells and treatment with Notch1 γ-secretase inhibitor prevented activation of Akt/mTOR. In addition, suppression of mTOR activity by rapamycin decreased the level of activity of p70S6K, induced upregulation of p53 and caspase 3, and led to increase of apoptosis in K562R^IMT^ cells. Inhibition of autophagy by spautin-1 or beclin-1 knockdown decreased exosomal release, but did not affect apoptosis in K562R^IMT^ cells. In summary, in K562R^IMT^ cells dasatinib promoted apoptosis through downregulation of Akt/mTOR activities, while preventing exosomal release and inhibiting autophagy by downregulating expression of beclin-1 and Vps34. Our findings reveal distinct dasatinib-induced mechanisms of apoptotic response and exosomal release in imatinib-resistant CML cells.

## 1. Introduction

Chronic myeloid leukemia (CML) is characterized by the uncontrolled proliferation of myeloid cells [1]. These leukemic cells contain a characteristic t (9:22) translocation resulting in the fusion of the Abelson (ABL) oncogene to the breakpoint cluster region (BCR) gene, and thus express a constitutively activated fusion protein BCR-ABL1, a tyrosine kinase, that is involved in the pathogenesis of CML [1]. The application of imatinib mesylate (IMT), an ATP-competitive selective BCR-ABL tyrosine kinase inhibitor, significantly improved survival in most CML patients [2]. However, not all patients present an effective response to imatinib. The most-studied mechanisms of imatinib resistance involve point mutations within ABL1 kinase domain and overexpression of BCR-ABL1 [3,4]. Additionally, research has also disclosed a number of BCR-ABL1-independent mechanisms such as overproduction of the ATP-binding cassette (ABC) transporter ABCG1 and ABCG2, upregulation of Src kinases, low activity or expression level of organic cation transporter (OCT-1), and presence of quiescent stem cells (e.g., primitive leukemic CD^34+^CD38^−^ cells) in some patients with imatinib treatment failure [3,4]. The resistance to imatinib led to the development of dasatinib, the second generation of tyrosine kinase inhibitors, showing enhanced inhibitory potency toward BCR-ABL1 [2]. In the clinic, dasatinib has shown superior efficacy in CML patients who developed BCR-ABL1 kinase mutations and showed resistance to imatinib therapy [2]. Although dasatinib could reduce cell growth and induce cell apoptosis in imatinib-resistant CML cells [5], treatment with the second-generation tyrosine kinase inhibitors may at times fail due to resistance or intolerance. Therefore, further investigation is still required to reveal the underlying mechanisms of tyrosine kinase inhibitor resistance in CML patients.

The mammalian target of rapamycin (mTOR) is a key pathway in cell growth and homeostasis, and its deregulation is involved in many diseases including cancer, cardiovascular disease, and diabetes [6]. The mTOR activity is regulated by the phosphoinositide 3-kinase (PI3K)/protein kinase B (Akt)/signaling pathway [7]. In many cancers, the PI3K/Akt/mTOR pathway is overactive, preventing apoptosis and supporting uncontrollable proliferation [8]. Both in murine myeloid 32D cells stably transformed with BCR-ABL (32D/BCR-ABL cells) and in K562 cells (a human BCR-ABL^+^ CML cell line), it was found that BCR-ABL1 kinase activated the PI3K/Akt signaling cascades, and abolished the repression of forkhead box protein O4 (FOXO4) on activating transcription factor 5 (ATF5), leading to activation of mTOR and then inhibition of the autophagic pathway [9]. Autophagy is used by the cells to degrade or remove unnecessary intracellular constituents including both proteins and organelles, also by means of releasing them to the extracellular environment as exosomes [10]. Recently, it was reported that CML cells could release exosomes, and that the addition of these vesicles to vascular endothelial cells, as well as to bone marrow stromal cells, may affect leukemia progression [11,12]. It was also shown that imatinib treatment can increase autophagic activity by inhibition of the PI3K/Akt/mTOR pathway [9]. Moreover, inhibition of autophagy by spautin-1 has been shown to enhance imatinib-induced apoptosis in K562 cells [13]. The inhibition of PI3K and Src kinase showed a synergistic effect with imatinib on induction of apoptosis and autophagy in K562 and in the cultured primary CML cells [14]. In the BCR-ABL^+^ LAMA cells and in the cultured primary leukemia cells, imatinib treatment led to activation of the PI3K/Akt/mTOR pathway [15]. Inconsistent Akt overactivation at Ser473 was also detected in imatinib-resistant CML patients, and imatinib resistance was decreased by mTOR inhibition only if Akt^Ser473^ was strongly activated [15]. However, the mechanism by which the PI3K/Akt/mTOR pathway is activated is not clear in imatinib-resistant CML cells.

In the present study, imatinib-resistant CML cells were produced from K562 after exposure to progressively increased concentrations of imatinib. The effects of the PI3K/Akt/mTOR signaling and autophagy activity on cellular apoptosis and exosomes release, as well as the potential efficacy of dasatinib were investigated in imatinib-resistant K562 cells (K562R^IMT^). We provided evidence that dasatinib promotes cellular apoptosis through inhibition of Akt/mTOR activities, and prevents exosomal release through downregulation of beclin-1 and Vps34 -dependent autophagic activity, indicating distinct dasatinib-induced mechanisms of apoptotic response and exosomes release in imatinib-resistant CML cells.

## 2. Results

### 2.1. Exosomes Release in K562R^IMT^ Cells

**Figure 1:**
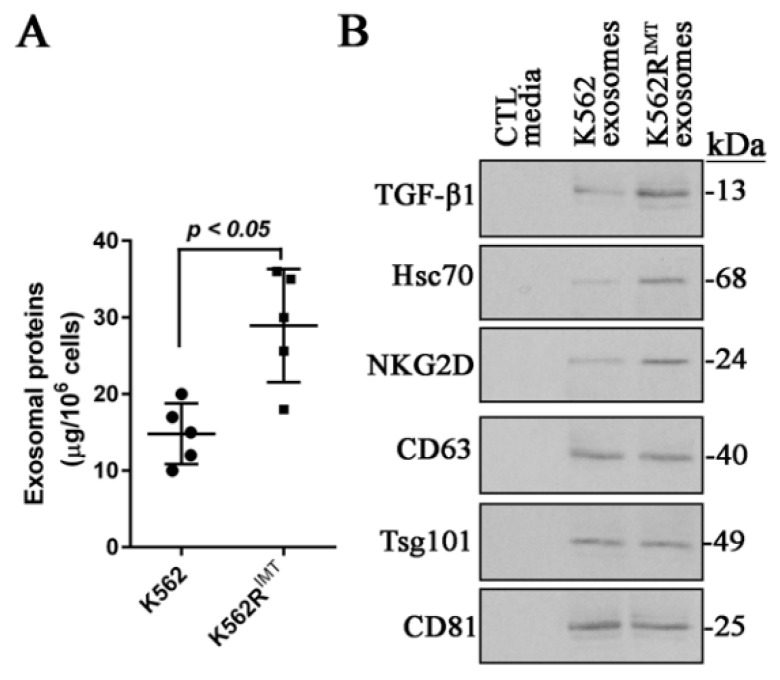
More exosomes are released from K562R^IMT^ cells. Exosomes were isolated from the cultured media of K562 and K562R^IMT^ cells, respectively. (**A**) BCA assay shows that the total amount of exosomal proteins from K562R^IMT^ was significant higher than that from K562. Data are shown as mean ± standard deviation (SD). *n* = 5 replicate experiments; (**B**) The exosomal proteins from 5 replicate experiments were equally pulled together. Totally, 100 µg each group was used for immunoblot of TGF-β1, Hsc70, and NKG2D as well as other exosomal markers CD63, Tsg101, and CD81. Culture media alone was used as negative control. As compared with K562, increased abundance of exosomal TGF-β1, Hsc70, and NKG2D was detected in K562R^IMT^ cells.

It has been shown that CML cell lines such as K562 and LAMA84 could release exosomes that may play an important role in modulating the tumor microenviroment and promoting angiogenesis [11,12,16]. Imatinib-resistant K562 cell line (K562R^IMT^) was produced by treating K562 cells with imatinib in a stepwise increased concentration, and exosomes were isolated in the culture media from K562 and K562R^IMT^ cells, respectively. BCA assay showed that the amount of total exosomal proteins from K562R^IMT^ was higher (*p* < 0.05) than that from K562 cells (Figure 1A). It was reported that TGF-β1, heat shock cognate protein 70 (Hsc70), and natural-killer group 2, member D (NKG2D) are present in exosomes released from K562 cells [12,16,17]. In the present study, TGF-β1, Hsc70 and NKG2D were also detected by using immunoblot assay in the isolated exosomal fractions from the media of K562 and K562R^IMT^ cells. Interestingly, the amounts of TGF-β1, Hsc70, and NKG2D were significantly higher in K562R^IMT^ exosomes compared to K562 exosomes, whereas other exosomal markers such as CD63, tumor susceptibility 101 (Tsg101) and CD81 showed no obvious difference between K562 and K562R^IMT^ cells (Figure 1B).

### 2.2. Activity of mTOR and Autophagy Is Increased in K562R^IMT^ Cells

**Figure 2:**
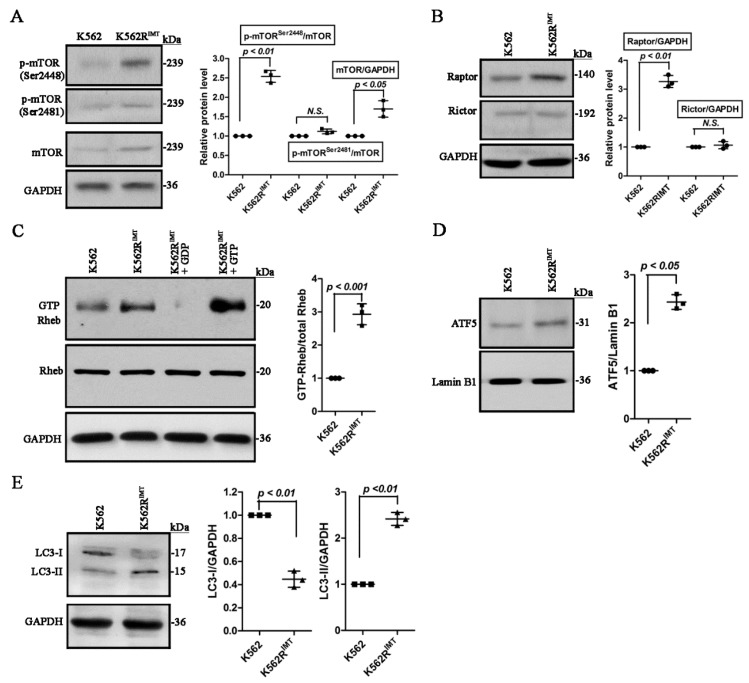
Activities of mTOR and autophagy are enhanced in K562R^IMT^ cells. Total cellular protein and nuclear protein of K562 and K562R^IMT^ cells was extracted by using RIPA lysis buffer and Nuclear Extraction Kit, respectively. (**A**) Immunoblot of total mTOR and phospho-mTOR at Ser2481 or Ser2448; (**B**) Immunoblot of two distinct mTOR complex markers Raptor and Rictor; (**C**) The level of activated Rheb. GTP-bound Rheb was immunoprecipitated by incubating cellular lysates with the specific mouse anti-active Rheb antibody and Protein A/G agarose and detected by using immunoblot with rabbit anti-Rheb antibody. GDP- or GTPγs-treated K562R^IMT^ lysates were used as the negative or positive control, respectively; (**D**) Immunoblot of the transcription factor ATF5 in nuclear fractions; (**E**) Immunoblot of different cleaved forms LC3-I and LC3-II of the autophagy marker LC3. Data are shown as mean ± SD. *n* = 3 independent experiments. *N.S.*: non significance.

The mammalian target of rapamycin (mTOR), is a key signaling pathway in cell growth and homeostasis, and was shown to be abnormally regulated in tumors [8]. The mTOR is phosphorylated at Ser^2448^ via the PI3 kinase/Akt signaling pathway and also autophosphorylated at Ser^2481^ [8]. Immunoblot assay showed that the relative abundance of total mTOR protein was significantly (*p* < 0.05) higher in K562R^IMT^ than K562 cells. Moreover, the level of phosphorylated mTOR at Ser^2448^ was increased significantly (*p* < 0.01) in K562R^IMT^ as compared with K562 cells. Remarkable difference was not detected for phospho-mTOR at Ser^2481^ between K562 and K562R^IMT^ cells (Figure 2A).

The mTOR functions in two distinct complexes. Raptor is a major component of mTOR complex1 (mTORC1) that regulates cell growth, survival, and autophagy, while Rictor is specific marker for mTOR complex 2 (mTORC2) that promotes cellular survival by activating Akt, regulates cytoskeletal dynamics via activating PKCα, and that controls ion transport and growth via SGK1 phosphorylation [8]. Here, upregulation of Raptor expression was shown (*p* < 0.01) in K562R^IMT^ cells in comparison with K562 (Figure 2B), implying that mTORC1 activity was increased in K562 cells following imatinib resistance development.

The small GTPase Rheb, in its GTP-bound state, is a necessary and potent stimulator of mTORC1 activity [8]. Consistently, the level of GTP-bound Rheb was significantly higher (*p* < 0.001) in K562R^IMT^ than K562 cells (Figure 2C). It was reported that mTOR may be a target of ATF5, or activating transcription factor 5 [9]. As compared with K562, the protein level of ATF5 increased significantly (*p* < 0.05) in K562R^IMT^ cells (Figure 2D), which may be responsible for the overproduction of the total mTOR protein.

Usually, mTOR plays a crucial role in regulating/inhibiting autophagy [18]. Immediately following synthesis, autophagy Light Chain 3 (LC3) is cleaved at the carboxy terminus and yields the cytosolic LC3-I form. During autophagy, LC3-I is converted to LC3-II through lipidation that allows for LC3 to become associated with autophagic vesicles [18]. The presence of LC3 in autophagosomes and the conversion of LC3-I to LC3-II have been used as indicators of autophagy [8,18]. LC3-II increased significantly (*p* < 0.01), whereas LC3-I decreased (*p* < 0.01) in K562R^IMT^ as compared with K562 cells (Figure 2E), indicating that mTOR-independent autophagy pathway is activated in K562R^IMT^ cells.

### 2.3. Induction of mTOR-Independent Autophagy Increases Exosomes Release in K562R^IMT^ Cells

**Figure 3:**
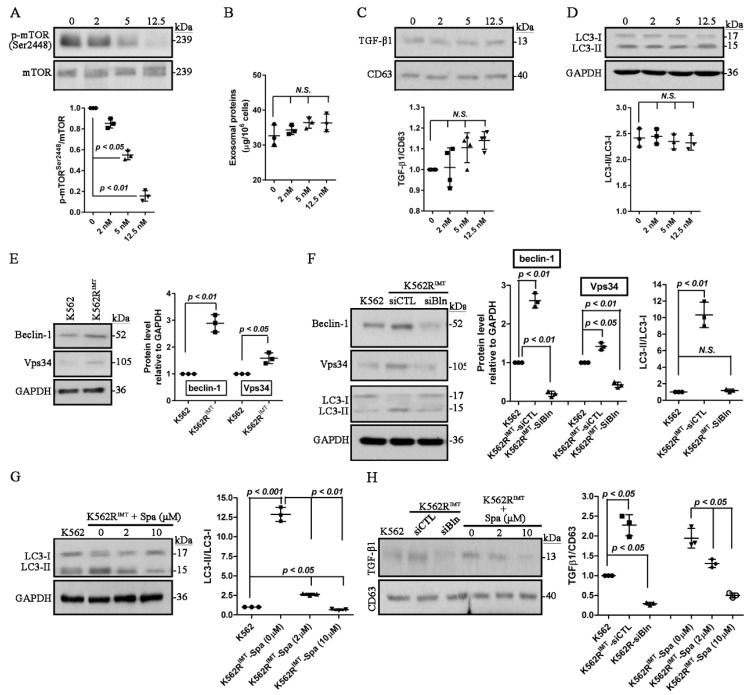
Inhibition of mTOR-independent beclin-1/Vps34 signaling decreases autophagy and exosomal release in K562R^IMT^ cells. (**A**–**D**) K562R^IMT^ cells were treated for 12 h with the mTOR inhibitor rapamycin. (**A**) Immunoblot assay shows that rapamycin dose-dependently decreased phospho-mTOR level at Ser2448 in K562R^IMT^ cells; (**B**) Exosomes were isolated from the cultured media of K562R^IMT^ cells, and total exosomal protein was determined using BCA assay. Rapamycin showed no effect on the amount of exosomal proteins in K562R^IMT^ cells; (**C**) Immunoblot of TGF-β1 and CD63 in the isolated exosomes. The abundance of TGFβ1 was not influenced by rapamycin application; (**D**) Immunoblot assay shows that rapamycin treatment did not affect the level of LC3-I and LC3-II in K562R^IMT^ cells; (**E**) As compared with K562, the abundance of beclin-1 and Vps34 increased significantly in K562R^IMT^ cells; (**F**) Knockdown of beclin-1 was performed in K562R^IMT^ cells by introduction of siRNA targeting to beclin-1 (siBln). The scrambled siRNA was used as control (siCTL). After 24 h, immunoblotting was used to evaluate the level of beclin-1, Vps34, and LC3 expression. In K562R^IMT^ cells, increased LC3-II and Vps34 was prevented significantly by beclin-1 knockdown; (**G**) K562R^IMT^ cells were treated for 12 h with the autophagy inhibitor spautin-1, and expression level of LC3 was analyzed using immunoblot assay. Increased LC3-II was inhibited significantly by spautin-1 in K562R^IMT^ cells; (**H**) Immunoblot of TGF-β1 in the isolated exosomes from K562 and K562R^IMT^ cells treated with spautin-1 or siBln. Beclin-1 knockdown or spautin-1 treatment prevented increase of exosomal TGFβ1 in K562R^IMT^ cells. Data are shown as mean ± SD. *n* = 3 independent experiments. *N.S.*: non significance.

Rapamycin is widely used as an inhibitor of mTORC1 signaling [18]. K562R^IMT^ cells were treated with rapamycin, and immunoblot assay showed that rapamycin downregulated (*p* < 0.05) the level of phospho-mTOR at Ser^2448^ in a dose-dependent manner (Figure 3A). Exosomes were isolated from the cultured media of K562R^IMT^ cells, and the total exosomal protein and exosomal TGF-β1 abundance were analyzed, respectively. Our data displayed that rapamycin treatment did not influence the amount of total exosomal protein (Figure 3B), and also showed no effect on the abundance of exosomal TGF-β1 in K562R^IMT^ cells (Figure 3C). Moreover, immunoblot assay showed that rapamycin treatment did not affect the conversion of LC3-I to LC3-II in K562R^IMT^ cells (Figure 3D). Our results suggested that mTORC1 inhibition by rapamycin showed no effects on exosomes release and autophagic activity in K562R^IMT^ cells.

Vps34, a member of the phosphatidylinositol 3-/4 (PI3/PI4)-kinase family, plays an important role in the regulation of mTOR protein synthesis, and also forms a complex with beclin-1 that promotes autophagy and tumor suppression [19]. Therefore, the protein level of beclin-1 and Vps34 was evaluated by using immunoblot assay in K562R^IMT^ cells. Our data showed that beclin-1 and Vps34 both increased significantly (*p* < 0.05) in K562 cells following imatinib resistance development (Figure 3E). To investigate the role of beclin-1 and Vps34 in regulating autophagic activity, silence of beclin-1 was obtained by using the specific siRNA. In K562R^IMT^ cells, knockdown of beclin-1 significantly (*p* < 0.05) decreased the protein level of Vps34 and LC3-II (Figure 3F), implying that upregulation of beclin-1 and Vps34 may be responsible for the increased autophagic activity in K562R^IMT^ cells.

Spautin-1, a very specific and potent autophagy inhibitor in mammalian cells, can promote degradation of Vps34 complexes and block the pro-survival autophagy pathway in cancer cells [20]. In K562R^IMT^ cells, the conversion of LC3-I to LC3-II was prevented significantly (*p* < 0.01) by spautin-1 application in a dose-dependent manner (Figure 3G). The effect of autophagy inhibition on exosomes release was further explored in K562R^IMT^ cells. The abundance of exosomal TGF-β1 was also decreased significantly (*p* < 0.05) in K562R^IMT^ cells by either beclin-1 knockdown or spautin-1 treatment (Figure 3H).

### 2.4. Loss of PTEN by Notch1 Activation Increases mTOR Activity in K562R^IMT^ Cells

**Figure 4:**
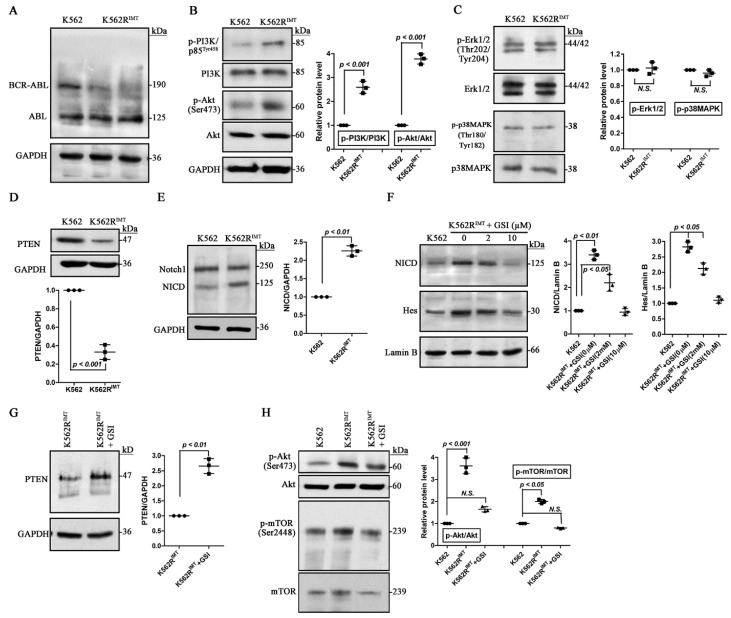
Loss of PTEN by Notch1 activation increases mTOR activity in K562R^IMT^ cells. Total cellular protein and nuclear protein was extracted by using RIPA lysis buffer and Nuclear Extraction Kit, respectively. Immunoblot assay was then performed. (**A**) Compared to K562, The BCR-ABL fusion protein level decreased significantly in K562R^IMT^ cells; (**B**) Abundance of phospho-PI3K/p85^Tyr458^ and phospho-Akt^Ser473^ was obviously higher in K562R^IMT^ than K562 cells; (**C**) The level of phospho-Erk1/2^Thr202/Tyr204^ and phospho-p38MAPK^Thr180/Tyr182^ showed no difference between K562 and K562R^IMT^ cells; (**D**) Expression level of PTEN inK562R^IMT^ cells was lower than that in K562; (**E**) Increased Notch 1 intracellular domain (NICD) was detected in K562R^IMT^ cells; (**F**) K562R^IMT^ cells were treated for 12 h with Notch γ-secretase inhibitor (GSI, RO4929097). Abundance of NICD and transcription factor Hes-1 was evaluated in nuclear fractions. GSI treatment dose-dependently prevented increase of NICD and Hes-1 in K562R^IMT^ cells; (**G**) Effect of GSI (10 µM) on PTEN expression level, showing that GSI increased PTEN level in K562R^IMT^ cells; (**H**) Effect of GSI (10 µM) on phospho-Akt^Ser473^ and phospho-mTOR^Ser2448^ levels. In K562R^IMT^ cells, increased phospho-Akt^Ser473^ and phospho-mTOR^Ser2448^ was inhibited by GSI application. Data are shown as mean ± SD. *n* = 3 independent experiments. *N.S.*: non significance.

PI3K and Akt is a major pathway that activates mTOR signaling [7,8]. In CML, BCR-ABL1 can promote cell survival by activating the PI3K/Akt pathway [21]. In the current study, we found that expression level of BCR-ABL1 was downregulated significantly in K562R^IMT^ compared to K562 cells (Figure 4A). Nevertheless, the level of phosphorylated PI3K/p85^Tyr458^ and Akt^Ser473^ increased significantly (*p* < 0.001) in K562R^IMT^ compared to K562 cells (Figure 4B). The level of phospho-MAPK^Thr180/Tyr182^ and phospho-Erk1/2^Thr202/Tyr204^ showed no difference between K562 and K562R^IMT^ cells (Figure 4C), indicating a specific involvement of the PI3K/Akt pathway activation in the development of imatinib resistance. The PI3K/Akt pathway is antagonized by various factors including PTEN, glycogen synthase kinase-3 beta (GSK3β), and homeobox protein 9 (HB9) [22]. Unlike most of the protein tyrosine phosphatases, PTEN preferentially dephosphorylates phosphoinositide substrates, and functions as a tumor suppressor by negatively regulating PI3K/Akt activation [22]. In K562R^IMT^ cells, PTEN expression level was decreased significantly (*p* < 0.001) (Figure 4D). It has been shown that Hes-1 could repress PTEN transcription downstream of Ras and Notch1 activation [23]. Notch pathway, a highly conserved cellular signaling system, involves diverse gene regulation mechanisms, and is dysregulated in many cancers [24]. Upon the ligand such as jagged-1 activation, Notch1 intracellular domain (NICD) produced by γ-secretase cleavage is released and transported to the nucleus thus forming a transcriptional activator complex with Rbpj and activating target gene transcriptions [24]. Immunoblot showed an obvious (*p* < 0.05) increase of both cytosolic and nucleic NICD in K562R^IMT^ cells as compared with K562 (Figure 4E,F). The specific γ-secretase inhibitor (GSI; RO4929097) was used to block Notch function. In the nuclear fraction from K562R^IMT^ cells, NICD and Hes-1 level was dose-dependently (*p* < 0.05) decreased by GSI application (Figure 4F). In addition, GSI significantly (*p* < 0.01) upregulated the expression level of PTEN in K562R^IMT^ cells (Figure 4G). The increased phospho-Akt^Ser473^ and phospho-mTOR^Ser2448^ level was significantly decreased (*p* < 0.05) by GSI treatment in K562R^IMT^ cells (Figure 4H). These results demonstrated that mTOR activation is mediated by the PI3K/Akt pathway through Notch-induced inhibition of PTEN in K562R^IMT^ cells.

### 2.5. Suppression of mTOR, Not Autophagic Activity, Increases Apoptosis in K562R^IMT^ Cells

**Figure 5:**
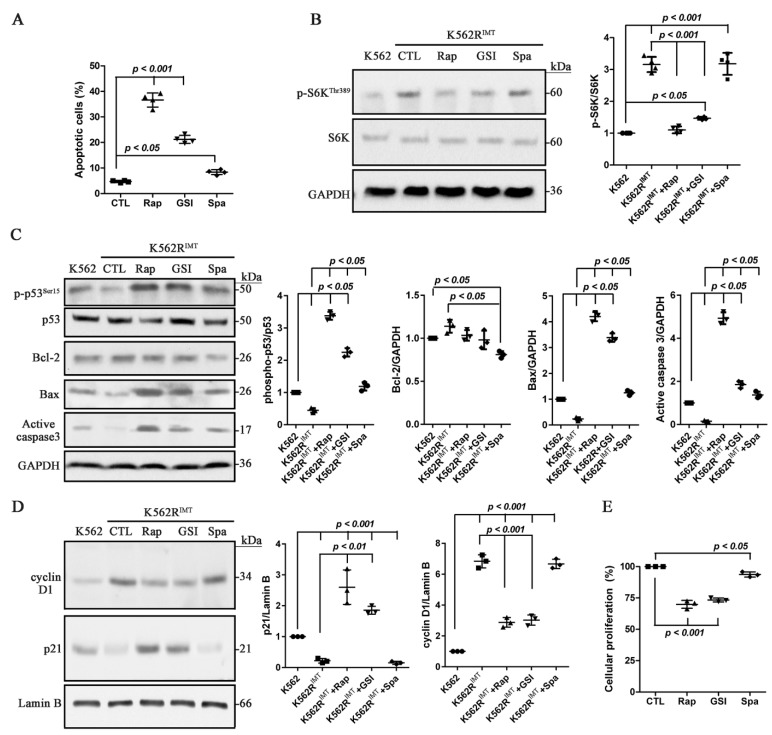
Suppression of mTOR activity, not autophagy increases apoptosis in K562R^IMT^ cells. K562R^IMT^ cells were treated for 12 h with rapamycin (Rap, 5 nM), Notch1 γ-secretase inhibitor (GSI, RO4929097; 10 µM) or spautin-1 (Spa, 2 µM). (**A**) Apoptosis assay showed that compared to control, rapamycin and GSI significantly induced apoptosis in the K562R^IMT^ cell; (**B**) Abundance of phospho-p70S6K^Thr389^ was analyzed using immunoblot assay, showing that increased phospho-p70S6K in K562R^IMT^ cell was prevented by rapamycin and GSI; (**C**) Immunobot assay was performed for expression of pro-apoptotic proteins phospho-p53^Ser15^, Bax and active caspase 3 as well as anti-apoptotic protein Bcl-2. In K562R^IMT^ cells, reduction of phospho-p53^Ser15^, Bax and activated caspase 3 was inhibited by rapamycin and GSI. Bcl-2 level showed no difference between K562 and K562R^IMT^ cells. Spautin-1 treatment only decreased Bcl-2 level in K562R^IMT^ cells; (**D**) Effect of rapamycin, GSI and spautin-1 on cell cycle proteins Cyclin D1 and p21 was evaluated using immunoblot assay. In K562R^IMT^ cells, cyclin D1 increased while p21 decreased, both of which were prevented by rapamycin and GSI; (**E**) Cellular proliferative ability was assessed using MTT assay in K562R^IMT^ cells, showing that the percentage of cellular proliferation was decreased by rapamycin and GSI. Data are shown as mean ± SD. *n* = 3 or 4 independent experiments.

The mTOR signaling promotes cellular survival possibly by inhibition of apoptosis through downstream signaling p70S6K-induced p53 blockage [25]. The effect of rapamycin, GSI and spautin-1 on cellular apoptosis was explored in K562R^IMT^ cells by using flow cytometry. Compared to spautin-1, apoptosis was induced significantly (*p* < 0.001) by rapamycin and GSI treatment (Figure 5A). As compared with K562, the phosphorylated p70S6K level at Thr389 increased significantly (*p* < 0.001) in K562R^IMT^ cells, which were significantly inhibited (*p* < 0.001) by mTOR inhibitor rapamycin and Notch1 inhibitor GSI, but not by autophagy inhibitor spautin-1 (Figure 5B). The level of apoptotic proteins phospho-p53^Ser15^, Bax, and the activated caspase 3 was significantly (*p* < 0.05) lower in K562R^IMT^ than that in K562 cells. As compared with spautin-1, GSI and rapamycin significantly (*p* < 0.05) increased the level of phospho-p53^Ser15^, Bax, and the activated caspase 3 in K562R^IMT^ cells (Figure 5C). Bcl-2 level showed no difference between K562 and K562R^IMT^ cells. Spautin-1 treatment slightly decreased Bcl-2 level in K562R^IMT^ cells (Figure 5C). Our results implied that mTOR, not autophagy pathway, plays a predominant role in regulation of apoptotic signaling in K562R^IMT^ cells.

Additionally, the role of mTOR and autophagy pathway on cellular proliferation was also investigated. Cyclin D1, a protein required for progression through the G1 phase of the cell cycle, has been found to be overproduced in some cancer cells [26]. In comparison, p21, a potent cyclin-dependent kinase inhibitor, functions as a regulator of cell cycle progression at G1 and S phase [26]. As compared with K562, overproduced cyclin D1 and decreased p21 was significantly (*p* < 0.05) detected in K562R^IMT^ cells, which was prevented (*p* < 0.01) by rapamycin and GSI, but not by spautin-1 (Figure 5D). MTT cellular proliferation assay showed that rapamycin and GSI significantly (*p* < 0.05) decreased the percentage of proliferative K562R^IMT^ cells (Figure 5E).

### 2.6. Dasatinib Enhances Apoptosis by Preventing mTOR Activation via Targeting Akt Pathway in K562R^IMT^ Cells

**Figure 6:**
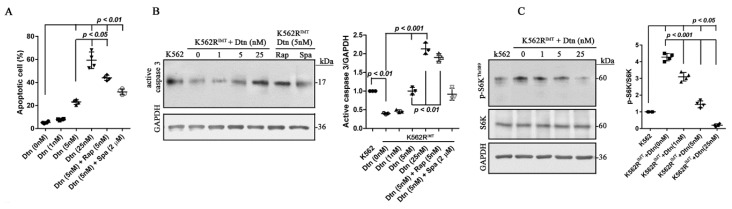

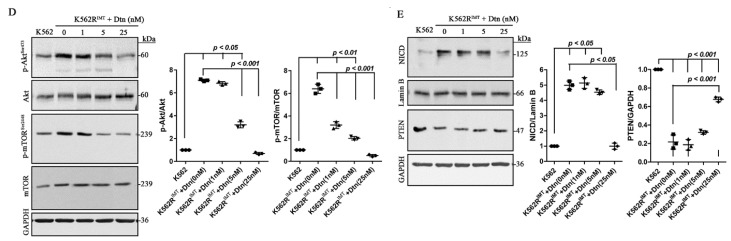
Dasatinib enhances apoptosis by inhibiting mTOR activation in K562R^IMT^ cells. K562R^IMT^ cells were treated for 24 h with dasatinib (Dtn) at different concentrations. Alternatively, K562R^IMT^ cells were pretreated for 12 h with rapamycin (5 nM) or spautin-1 (2 µM), and then treated for 24 h with dasatinib (5 nM). (**A**) Percentage of apoptotic cells was evaluated by using flow cytometry. Dasatinib dose-dependently induced apoptosis of K562R^IMT^ cells. The synergistic effect on cellular apoptosis was observed in dasatinib and rapamycin treated cells; (**B**) The level of activated caspase 3 was analyzed using immunoblot assay, showing that dasatinib dose-dependently increased the level of active caspase 3 in K562R^IMT^ cells. Rapamycin enhanced the effect of dasatinib on caspase 3 in K562R^IMT^ cells; (**C**) Immunoblot assay showed that dasatinib dose-dependently decreased the level of phospho-p70S6K^Thr389^ in K562R^IMT^ cells; (**D**) Effect of dasatinib on the level of phospho-Akt^Ser473^ and phospho-mTOR^Ser2448^. Immunoblot assay shows that dasatinib dose-dependently decreased phospho-Akt^Ser473^ and phospho-mTOR^Ser2448^ level in K562R^IMT^ cells; (**E**) Effects of dasatinib on the level of Notch1 intracellular domain (NICD) and PTEN were evaluated by using Immunoblot assay, showing that increased NICD was decreased in a dose-dependent manner in K562R^IMT^ cells. High dose of dasatinib (25 nM) prevented reduction of PTEN in K562R^IMT^ cells. Data are shown as mean ± SD. *n* = 3 or 4 independent experiments.

It has been shown that the second-generation tyrosine kinase inhibitor dasatinib functions well in the CML patients with imatinib resistance [4]. However, the underlying mechanism has notbeen fully clarified. In dasatinib-treated K562R^IMT^ cells, the percentage of apoptotic cell increased significantly (*p* < 0.01) in a dose-dependent manner (Figure 6A). Inhibition of mTOR signaling with rapamycin, not autophagy inhibition by spautin-1, significantly (*p* < 0.05) enhanced the efficacy of dasatinib on induction of cellular apoptosis in K562R^IMT^ cells (Figure 6A). Consistently, dasatinib treatment significantly (*p* < 0.05) increased the level of the activated caspase 3, which was enhanced (*p* < 0.01) by rapamycin, not by spautin-1 in K562R^IMT^ cells (Figure 6B). Furthermore, dasatinib dose-dependently prevented increase of phospho-p70S6K^Thr389^ in K562R^IMT^ cells (Figure 6C). The potential target of dasatinib on cellular apoptosis was further investigated. In K562R^IMT^ cells, dasatinib significantly (*p* < 0.05) decreased the level of phospho-Akt^Ser473^ and phospho-mTOR^Ser2448^ in a dose-dependent manner (Figure 6D), but only showed an obvious influence on the level of nuclear NICD and total PTEN at high concentrations (25 nM) (Figure 6E). Our results indicated that dasatinib may promote apoptosis by preventing mTOR activation predominantly through downregulation of Akt activation in K562R^IMT^ cells.

### 2.7. Dasatinib Decreases Exosome Release by Inhibiting Autophagy Activation in K562R^IMT^ Cells

**Figure 7:**
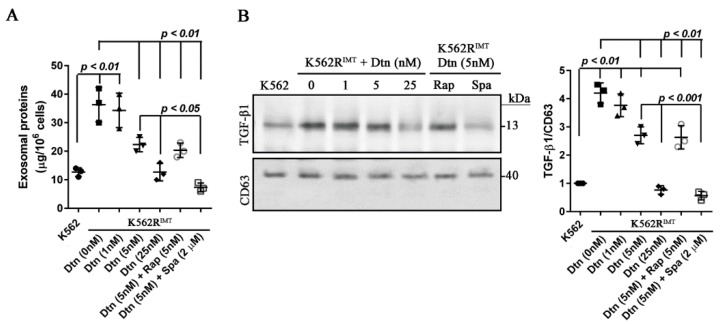

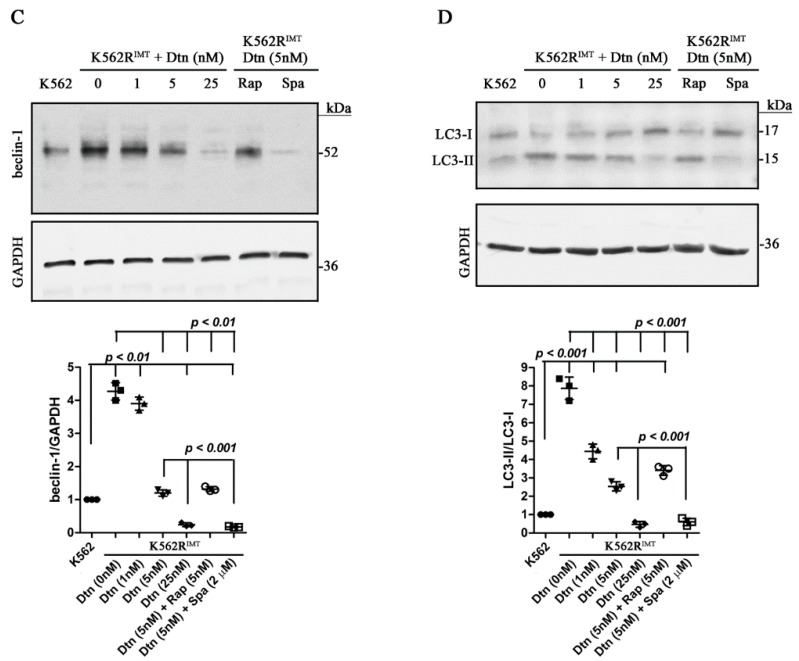
Dasatinib decreases exosome release by inhibiting autophagy activity in K562R^IMT^ cells. K562R^IMT^ cells were treated for 24 h with dasatinib (Dtn) at different concentrations. Alternatively, K562R^IMT^ cells were pretreated for 12 h with rapamycin (5 nM) or spautin-1 (2 µM), and then treated for 24 h with dasatinib (5 nM). (**A**) Exosomes were isolated, and total exosomal protein was determined using BCA assay. Compared to K562, the amount of exosomal proteins increased significantly in K562R^IMT^ cells, which was prevented by dasatinib in a dose-dependent manner and spautin-1 showed a synergistic effect; (**B**) Immunoblot of TGF-β1 in the isolated exosomes. Dasatinib dose-dependently decreased the abundance of TGF-β1 in K562R^IMT^ cells, and spautin-1 showed a synergistic effect; (**C**) The level of beclin-1 was analyzed using immunoblot assay. In K562R^IMT^ cells, dasatinib dose-dependently decreased the level of beclin-1, and spautin-1 showed a synergistic effect; (**D**) The levels of LC3-I and LC3-II were analyzed using immunoblot assay. Increased LC3-II level was dose-dependently prevented by dasatinib, and spautin-1 showed a synergistic effect. Data are shown as mean ± SD. *n* = 3 independent experiments.

Our data has shown that exosome release was increased in K562R^IMT^ cells. The effect of dasatinib on exosome release was thus explored in the current study. Total exosomal protein was dose-dependently (*p* < 0.05) decreased by dasatinib treatment in K562R^IMT^ cells, which was enhanced (*p* < 0.05) by spautin-1 administration, not by rapamycin (Figure 7A). The abundance of TGF-β1 in the isolated exosomes from the media of K562R^IMT^ cells was also significantly (*p* < 0.05) decreased by dasatinib treatment, and spautin-1 application, not rapamycin, showed a combined or synergistic effect (Figure 7B). Immunoblot assay showed that beclin-1 and LC3-II were also (*p* < 0.05) decreased by dasatinib, which was (*p* < 0.05) enhanced by spautin-1, not rapamycin (Figure 7C,D). Our results demonstrated that dasatinib decreases exosome release by inhibiting autophagy pathway activation through downregulation of beclin-1 in K562R^IMT^ cells.

## 3. Discussion

Overactivation of the PI3K/Akt signaling pathway induced by upregulation of the BCR-ABL level is a major factor for the development of imatinib resistance in CML [3,4,21]. In addition, frequencies of mutation in BCR-ABL kinase domain appear to increase and induce resistance to tyrosine kinase inhibitors as CML progresses [27]. It was reported that sequencing of the BCR-ABL kinase domain did not find mutations both in K562 and in K562R^IMT^ cells [28,29]. As Lee *et al.* [30] described, we also found that the BCR-ABL level decreased significantly in K562R^IMT^ cells (Figure 4A). Therefore, overactivation of PI3K/Akt in these cells is BCR-ABL-independent (Figure 4B and Figure 6D). The mTOR pathway is a major target of the PI3K and Akt signaling [8]. There are two distinct complexes of mTOR, mTORC1 and mTORC2, which are indicated by the specific markers Raptor and Rictor, and contain mTOR phosphorylated predominantly on Ser2448 and Ser2481, respectively [8,31]. GTP-bound Rheb, a small GTPase, is a potent stimulator of the mTORC1 activity responsible for activation of mTOR on Ser2448 [8]. In this study, increased phospho-mTOR^Ser2448^ and Raptor as well as increased active form of Rheb (Figure 2C) were significantly detected in K562R^IMT^ cells (Figure 2A,B), indicating that the mTORC1 is activated in imatinib-resistant CML cells. Additionally, our results suggested that upregulation of ATF5 (Figure 2D) may lead to an increase of total mTOR protein (Figure 2A) since ATF5 is a key transcription factor of mTOR. The mTOR signaling promotes cellular survival possibly by inhibition of apoptosis through downstream signaling p70S6K-induced p53 blockage [8]. In our study, increased phospho-p70S6K^Thr389^ but decreased apoptotic proteins including phospho-p53^Ser15^, Bax and active caspase 3 were detected in K562R^IMT^ cells, compared to K562 cells (Figure 5B,C).

Generally, autophagy pathway is negatively regulated by mTOR signaling [18]. Rapamycin inhibits mTOR pathway by directly binding to mTORC1 complex [32]. Our data demonstrated that the increased autophagy activity is mTOR-independent in imatinib-resistant CML cells (Figure 2E and Figure 3B–D). It is well known that autophagy activity can also be promoted by Vps34 by forming a complex with beclin-1 [20]. In this study, compared to K562, beclin-1 and Vps34 both increased significantly in K562R^IMT^ cells (Figure 3E). Consistently, infection of leukemia cells including K562 cells with adenovirus overexpressing beclin-1 enhanced autophagic activity [33]. Mounting evidence discloses a close relationship between the autophagy pathway and the biogenesis and secretion of exosomes [10]. It has been well known that exosomes have some specialized functions, either a beneficial or a detrimental impact on neighboring cells [34]. Exosome release has been reported in the human CML cell line K562 and LAMA84 cells [11,12,16,35,36]. In this study, we also found that K562 cells release or secrete many more exosomes when developing resistance to imatinib (Figure 1A,B). In K562 cells, it has been reported that spautin-1, a specific autophagy inhibitor, significantly blocked imatinib-induced autophagic activation by downregulating beclin-1 [13]. Our results also showed that spautin-1 treatment or beclin-1 knockdown prevented increase of autophagic activity and thus exosomal TGF-β1 release (Figure 3F–H). Therefore, our findings further demonstrated that the mTOR-independent beclin-1/Vps34 signaling may be responsible for induction of autophagy and exosomal release in K562R^IMT^ cells. Nevertheless, the molecular components and function of exosomes need be further examined in imatinib-resistant CML cells.

It has been well known that PTEN preferentially dephosphorylates phosphoinositide substrates, and functions as a tumor suppressor by negatively regulating the Akt signaling pathway [22]. We found that the PTEN protein level was much lower in K562R^IMT^ cells than that in K562 (Figure 4D). Dahia *et al.* also described that the abundance of PTEN both at mRNA and protein level was very low in three myeloid cell lines including K562, KU812, and U937, and that sequencing analysis showed no mutation of PTEN in these cell lines [37]. As the downstream target of Ras and Notch1 pathway, Hes-1 could repress PTEN transcription [23]. Notch pathway necessary for diverse gene regulation is dysregulated in many cancers [23,24]. Upon the ligand such as jagged-1 induced activation, notch intracellular domain (NICD) is produced through cleavage of γ-secretase, and then transported to the nucleus and forms a transcriptional activator complex with Rbpj [24]. An obvious increase of both cytosolic and nucleic NICD as well as nuclear Hes-1 was detected in K562R^IMT^ cells as compared with K562 (Figure 4E,F). RO4929097, the specific Notch γ-secretase inhibitor (GSI), effectively blocks Notch signaling activation [24]. In K562R^IMT^ cells, our results from GSI inhibition assay demonstrated that Notch1 activation-mediated downregulation of PTEN protein level may be responsible for Akt activation and mTOR activity induction (Figure 4F–H).

Furthermore, the effects of Notch1, mTOR and autophagy pathways on apoptosis induction were investigated by using their specific inhibitors in this study. Our data suggested that rapamycin and GSI treatment, not spautin-1 induced apoptosis by reducing phospho-p70S6K^Thr389^ (Figure 5B) and increasing apoptotic proteins in K562R^IMT^ cells (Figure 5C). In K562 cells, it was reported that spautin-1 enhanced imatinib-induced cell apoptosis by inactivating PI3K/Akt and activating its downstream protein GSK3β, leading to downregulation of the anti-apoptotic proteins Mcl-1 and Bcl-2, the downstream effectors of autophagic signaling [13]. In the present study, spautin-1 led to a slight reduction of Bcl-2 in K562R^IMT^ cells (Figure 5C), implying that inhibition of autophagic activity may play a minor role in induction of apoptosis in imatinib-resistant K562 cells. Additionally, our results also showed that rapamycin and GSI significantly decreased the proliferative ability of K562R^IMT^ cells (Figure 5D,E). Hence, our results implied that mTOR may play a predominant role in regulation of apoptotic and proliferative signaling in K562R^IMT^ cells. Nevertheless, it was reported that the activated Notch signaling by overexpression of NICD in K562 cells mildly but significantly inhibited cell proliferation and reduced the ability of colony formation, suggesting that the Notch signaling may function as a tumor inhibitor in human CML cells [38]. Therefore, further studies are needed to illustrate the function and mechanisms of Notch pathway in human CML, especially in imatinib resistant CML cells.

Rapamycin, not spautin-1, significantly enhanced the efficacy of dasatinib on induction of cellular apoptosis in K562R^IMT^ cells (Figure 6A,B). In K562R^IMT^ cells, dasatinib dose-dependently decreased phosphorylation of Akt^Ser473^, mTOR^Ser2448^ and p70S6K^Thr389^, but only showed a slight effect on NICD and PTEN at high concentrations (Figure 6C,D). Our results suggested that the Akt/mTOR/p70S6K/caspase 3 pathway may be involved in dasatinib-mediated growth suppression of K562R^IMT^ cells. Nevertheless, it should be further verified by overexpressing constitutively active p70S6K in K562R^IMT^ cells. Similarly, it was reported that the downregulation of the PI3K/Akt/mTORC1 signaling cascades may be a crucial mediator in the inhibition of proliferation and induction of apoptosis by resveratrol in K562 cells [39]. In addition, it was found that activation of p38MAPK signaling pathway is essential for the anti-leukemic effects of dasatinib [40], but we did not find obvious difference of phospho-p38MAPK^Thr180/Tyr182^ and phospho-Erk1/2^Thr202/Tyr204^ between K562 and K562R^IMT^ cells (Figure 4C). In several imatinib-resistant CML cell lines such as K562R, LAMA84R, and KCL22R, simvastatin, one of the most pharmacologically potent inhibitors of HMG-CoA reductase, was found to have a synergistic killing effect by induction of apoptosis and cell cycle arrest by inhibiting tyrosine phosphorylation and activating STAT5 and STAT3 [41]. Nilotinib is more potent than imatinib in inhibiting BCR-ABL tyrosine kinase activity and proliferation of BCR-ABL-overexpressing cells [42]. In K562 cells resistant to nilotinib, it was found that dasatinib induces apoptosis potentially by inhibiting Lyn kinase activity, downregulating cyclin D1 and upregulating p21 [43]. Therefore, the mechanism by which CML cells develop resistance following treatment with imatinib, and the mechanism of the killing effects of the novel anti-CML drugs, should be further investigated.

Total exosome release was reduced by imatinib and dasatinib in K562 cells [12]. In this study, we found that dasatinib decreased beclin-1 and LC3-II expression as well as exosomal release in a dose-dependent manner in K562R^IMT^ cells, which were enhanced by spautin-1, not by rapamycin (Figure 7). Our results implied that dasatinib decreased exosome release by inhibiting autophagy activity by downregulating beclin-1 in K562R^IMT^ cells. Additionally, it was reported that Rab11 and VAMP3 are required for the fusion between multivesicular bodies (MVBs) with autophagosomes to allow the maturation of the autophagosome, while VAMP7 and ATPase NSF, a protein required for SNAREs disassembly, participate in the fusion between MVBs with the plasma membrane to release exosomes into the extracellular medium [44].

**Figure 8:**
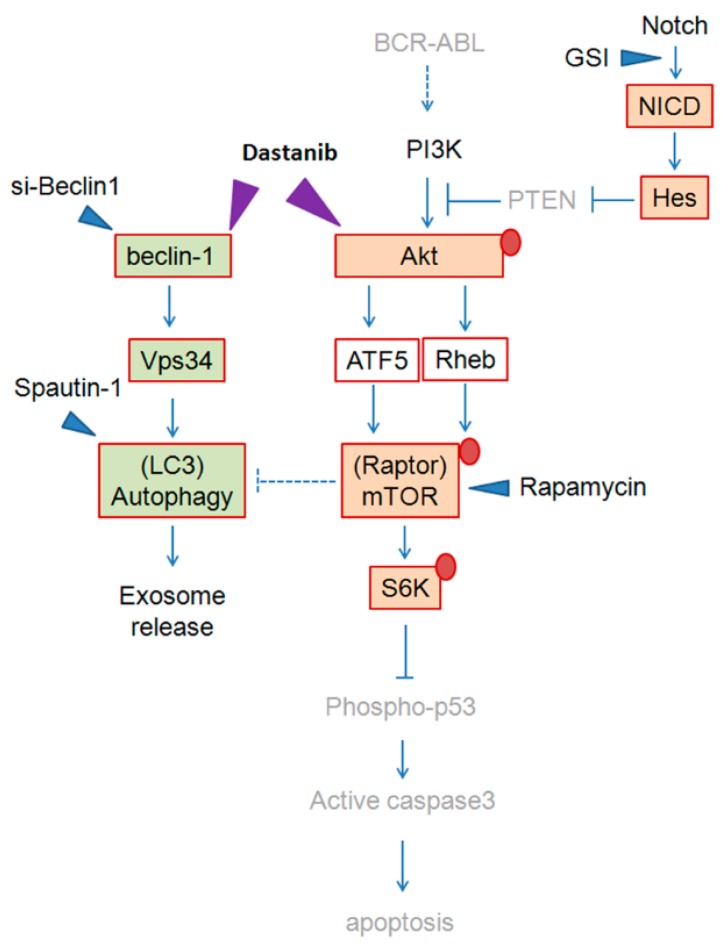
The schematic of proposed signaling pathways involved in imatinib resistance and dasatinib target.

Altogether, our study indicated a distinct role of mTOR signaling and autophagic pathway in imatinib-resistant K562 cells, being responsible for inhibition of apoptosis and induction of exosome release, respectively. Moreover, our data demonstrated that dasatinib promotes cellular apoptosis through downregulation of Akt/mTOR activity, and prevents exosome release by inhibiting beclin-1/Vps34-depedndent autophagic activity (Figure 8). Notably, we only used K562R^IMT^ cells in the current study, and a variety of imatinib-resistant CML cell lines need be examined to understand well the mechanisms of the killing effects of dasatinib in CML patients.

In imatinib-resistant K562 cells, BCR-ABL level is decreased. Reduction of PTEN via Notch/Hes-1 signaling leads to the PI3K/Akt/mTOR pathway activation, which is responsible for upregulation of p-p70S6K^Thr389^ and thus inhibition of apoptosis. GSI and rapamycin, not spautin-1, results in induction of apoptosis. In addition, activation of autophagy through mTOR-independent beclin-1/Vps34 signaling enhances exosome release since beclin-1 knockdown and spautin-1 treatment, not rapamycin, prevents exosome release. Dasatinib induces p-p53^Ser15^/active caspase 3 expression and thus promotes apoptosis by inhibiting the mTOR/p70S6K^Thr389^ activity via downregulation of Akt^Ser473^ activation, and decreases exosomal release by inhibiting autophagy activity via downregulation of beclin-1 and Vps34. GSI: Notch1 γ-secretase inhibitor; Spautin-1: autophagy inhibitor; Rapamycin: mTOR inhibitor; si-Beclin-1: knockdown of beclin-1 by siRNA. Red circle: phosphorylation status.

## 4. Experimental Section

### 4.1. Antibodies

The primary antibodies used in this study were: rabbit anti-CD63, IL-17, γ-IFN, IL-10, Raptor, Rictor, beclin-1, cyclin D1, p21, Bcl-2, Erk1/2 or Bax, and mouse anti-PI3K, Akt, p-Erk1/2^Thr202/Tyr204^ or TGF-β1 (Abcam, Cambridge, MA, USA); rabbit anti-phospho-mTOR^Ser2481^, phospho-mTOR^Ser2448^, phopsho-PI3K/p85^Tyr458^, phospho-Akt^Ser473^ or cleaved caspase 3, c-ABL or p38MAPK, and mouse anti-NKG2D, mTOR, PTEN or phospho-p53^Ser15^ (Cell Signaling, Beverly, MA, USA); rabbit anti-ATF5 or phospho-p70S6K^Thr389^, and mouse anti-GAPDH (Sigma-Aldrich, St. Louis, MO, USA); rabbit anti-LC3 and mouse anti-Hes (NOVUS); rabbit anti-Hsc70 or p70S6K, and mouse anti-Notch1 (Pierce, Waltham, MA, USA); rabbit anti-Lamin B1 or p53, mouse anti-p-p38MAPK^Thr180/Tyr182^ (Santa Cruz, Dallas, TX, USA), and rabbit anti-Vps34 (Life Technologies, Carlsbad, CA, USA).

### 4.2. Cell Culture and Treatment

The CML cell line K562 (ATCC, Manassas, VA, USA) was cultured at 37 °C in DMEM containing 10% FBS (Gibco, Waltham, MA, USA) and 100 U/mL of Penicillin/Streptomycin. To make imatinib-resistant CML cell line, K562 was continuously exposed to higher concentrations of imatinib mesylate (IMT; Sigma-Aldrich, St. Louis, MO, USA) in stepwise increase of 100 nM from 0.1 to 1 µM after 7 days of culture [43]. Finally, the viable imatinib-resistant cells (K562R^IMT^) were maintained in culture media containing 1 µM of imatinib. To evaluate the effects of dasatinib on cellular apoptosis and exosomes release, K562R^IMT^ cells were treated with the indicated dosage of dasatinib (Sigma-Aldrich, St. Louis, MO, USA) for 24 h in the absence of imatinib. As Okabe *et al.* [45] reported, our preliminary results demonstrated that removal of imatinib for 24 hours showed no effects on cleaved caspase 3 and Akt activation at Ser473 in imatinib-resistant K562 cells.

In experiments using the mTOR inhibitor rapamycin (Sigma-Aldrich), Notch γ-secretase inhibitor GSI (RO4929097, Selleck, Houston, TX, USA), or autophagy inhibitor spautin-1 (Sigma-Aldrich), K562R^IMT^ cells were pretreated with these substances for 12 h.

### 4.3. Exosome Isolation

Culture media were ultra-centrifuged at 100,000× *g* for 16 h at 4 °C to remove exosomes that may be possibly present in media. K562 or K562R^IMT^ cells were cultured in a 150-cm^2^ flask for 24 h in ultra-centrifuged media, and then the cultured media were collected for exosome purification as described previously [12]. Briefly, the media were centrifuged progressively at 300× *g* for 10 min, 2000× *g* for 10 min, and then filtered through a 0.22 µm filter. Effluent was ultra-centrifuged at 100,000× *g* for 2 h. The exosomal pellets from 5 replicate experiments were resuspended in PBS, and then the content of exosomal protein was determined using BCA assay (Thermo Scientific, Waltham, MA, USA).

### 4.4. Knockdown of Beclin-1

K562R^IMT^ was cultured in a 6-well plate, and transfected with a small interfering RNA targeted to human beclin-1 (si-Bln: gctgccgttatactgttct) [46] or a scramble negative control (si-CTL: gttctccgaacgtgtcacgt) by using Lipofectamine RANiMAX reagent (Invitrogen, Grand Island, NY, USA). After 48 h, cells were lysed for Western blot analysis.

### 4.5. Western Blot

Total cellular protein was extracted with RIPA buffer containing freshly added protease and phosphotase inhibitor cocktail (Roche, Indianapolis, IN, USA). Nuclear protein was extracted using the Nuclear Extraction Kit (Abcam). Protein concentration was determined using BCA assay. Equal amounts of protein were separated on a 7.5% or 12% SDS gel and electrophoretically transferred to a nitrocellulose membrane. Membranes were blocked with 5% non-fat milk or BSA in Tris-buffered saline containing 0.05% Tween-20 (TTBS) for 1 h. The indicated primary antibody was incubated overnight at 4 °C. After washing three times with TTBS, membranes were incubated with HRP-conjugated goat anti-rabbit or mouse antibody (1:10,000; Thermo Scientific) for 1 h, and then developed with the enhanced chemiluminescence reagent (Thermo Scientific). The specific band was quantified using Image J software (NIH).

### 4.6. Rheb Activity Detection

The active Rheb-GTP level was measured with Rheb Activation Assay Kit (Abcam). Briefly, K562 or K562R^IMT^ cells were lysed with 1× Assay/Lysis Buffer supplemented with protease inhibitors (Roche). The anti-active Rheb mouse monoclonal antibody was incubated with 250 µg of cellular lysates (adjusted volume to 500 μL with 1× Assay/Lysis Buffer) at 4 °C overnight. The bound activated Rheb-GTP was then pulled down by incubating for 1 h with 25 µL of Protein A/G agarose at 4 °C. The agarose beads were washed 5 times by centrifugation (5000× *g*, 1 min) with 500 µL of 1× Assay/Lysis Buffer. The beads were then resuspended in 25 µL of 2× Lamelli loading buffer and boiled for 5 min. The precipitated active Rheb-GTP was detected by Western blot using rabbit anti-Rheb polyclonal antibody. Notably, the same amount of lysates from K562R^IMT^ cells were treated with 0.5 M EDTA (final 20 mM) and 100× GDP (final 1 mM; negative control) or 100× GTPγs (final 1 mM; positive control) for 30 min with agitation at room temperature, and then the reactions were stopped by putting the tubes on ice and adding 1 M MgCl_2_ (final 60 mM).

### 4.7. Cellular Proliferation Assay

Cell proliferation was measured using MTT Cell Proliferation Assay Kit according to the manufacture’s protocol (BioVision, Rockland, MA, USA).

### 4.8. Apoptosis Detection Assay

The cleaved caspase 3 was evaluated in live cells by using the APO ACTIVE 3™ Kit (Cell Technology, Fremont, CA, USA) as previously described [47]. Briefly, 1 × 10^6^ cells were fixed by incubation in 500 μL of 1× fixative solution at room temperature for 15 min. After washing twice with PBS, the cells were resuspended in 1 mL of 1% Saponin/PBS and 100 μL of cells were pipetted out into a 2-mL tube. After adding 10 μL of the 1× rabbit anti active caspase 3 antibody, they were incubated for 1 h at room temperature. After washing twice with 1% saponin/PBS, 10 μL of the 1× FITC labeled goat anti rabbit IgG was added and then the contents was incubated for 1 h at room temperature. After washing once with 1% Saponin/PBS and 2% BSA/PBS respectively, the cells were resuspended in 500 μL of 2% BSA/PBS. The caspase 3 positive cells were counted with flow cytometry (FACScan, BD, San Jose, CA, USA). Additionally, the level of the activated caspase 3 was also evaluated by using Western blotting with the specific anti-activated caspase 3 antibody.

### 4.9. Statistics

Data are shown as mean ± SD. Statistical analysis was performed with Prism 4 (GraphPad Software, La Jolla, CA, USA) using unpaired test or ONE-WAY ANOVA followed by Tukey’s multiple comparisons test. *p* ≤ 0.05 was regarded as significant differences.
